# How to Save a Drowning Person

**Published:** 1864-04

**Authors:** 


**How to Save a Drowning Person.**—It may not be generally known that when a person is drowning, if he is taken by the arm from behind, between the elbow and shoulder, he can not touch the person attempting to save him, and whatever struggles he may make will only assist the person holding him in keeping his head above water. A good swimmer can keep a man thus above water for an hour. If seized any where else the probability is that he will clutch the swimmer, and perhaps, as is often the case, both will be drowned.

